# Optimization of library preparation based on SMART for ultralow RNA-seq in mice brain tissues

**DOI:** 10.1186/s12864-021-08132-w

**Published:** 2021-11-10

**Authors:** Erteng Jia, Huajuan Shi, Ying Wang, Ying Zhou, Zhiyu Liu, Min Pan, Yunfei Bai, Xiangwei Zhao, Qinyu Ge

**Affiliations:** 1 State Key Laboratory of Bioelectronics, School of Biological Science & Medical Engineering, Southeast University, Nanjing, 210096 China; 2 School of Medicine, Southeast University, Nanjing, 210097 China

**Keywords:** scRNA-seq, Sensitivity, Template-switching oligos terminal modification, Low abundance gene detection, Subcellular

## Abstract

**Background:**

Single-cell RNA sequencing (scRNA-seq) provides new insights to address biological and medical questions, and it will benefit more from the ultralow input RNA or subcellular sequencing.

**Results:**

Here, we present a highly sensitive library construction protocol for ultralow input RNA sequencing (ulRNA-seq). We systematically evaluate experimental conditions of this protocol, such as reverse transcriptase, template-switching oligos (TSO), and template RNA structure. It was found that Maxima H Minus reverse transcriptase and rN modified TSO, as well as all RNA templates capped with m7G improved the sequencing sensitivity and low abundance gene detection ability. RNA-seq libraries were successfully prepared from total RNA samples as low as 0.5 pg, and more than 2000 genes have been identified.

**Conclusions:**

The ability of low abundance gene detection and sensitivity were largely enhanced with this optimized protocol. It was also confirmed in single-cell sequencing, that more genes and cell markers were identified compared to conventional sequencing method. We expect that ulRNA-seq will sequence and transcriptome characterization for the subcellular of disease tissue, to find the corresponding treatment plan.

**Supplementary Information:**

The online version contains supplementary material available at 10.1186/s12864-021-08132-w.

## Background

Single-cell RNA sequencing (scRNA-seq) technologies provide a unique opportunity to analyze the single-cell transcriptional landscape. It is a transformative technology that is rapidly deepening our understanding of biology [1, 2]. This technology can be used for unbiased assessment of cellular heterogeneity with high resolution and high accuracy, identify the subtypes of single cells, accurately determine the gene expression level of single cells, and explain genetic information heterogeneity in a comprehensive and multi-level manner at the single cell level. However, the limitations of scRNA-seq sensitivity and the associated transcript absence events (dropouts) limit cell clustering and the faithful delineation of cell subtypes, which hamper downstream analyses. Based on the full-length transcriptome sequencing, such as Smart-seq [3], Smart-seq2 [4], MATQ-seq [5], etc., the full-length transcripts can be detected, which improve the sensitivity and accuracy of gene detection, and perform various types of transcriptome sequencing, but this method has less cellular throughput and higher cost-efficiency. Based on the 3′ or 5′ ends of transcripts sequencing, such as Drop-seq [6], CEL-seq2 [7], Seq-Well [8], STRT-seq [9] etc., have high cellular throughput and low cost-efficiency. However, these methods only detect one end of transcripts and have low sensitivity to detect gene expression. So it is not suitable for the analysis of variable splicing, allelic, and low abundance genes. At present, scRNA-seq method is mainly applied to single cells or 10 pg total RNA samples [10, 11], but the subcellular sequencing scheme has not been studied. The mapping of different subcellular RNA maps provides a new perspective on studying the relationship between the dynamic regulation of RNA subcellular space and the occurrence of human diseases [12]. Although each scRNA-seq platform has their advantages, they suffer from low mRNA capture efficiency [6, 8, 13–15], and their sensitivity for detecting genes with low expression and coverage uniformity varies [16], which result in a loss of valuable information. This has an important impact on the detection of cancer-related mutant genes and important low-expressed genes in biology, such as transcription factors.

At present, the optimization of low mRNA capture efficiency and the sensitivity of detecting low-abundance expressed genes is mainly from two aspects: scRNA-seq technology and data analysis. Previous studies have shown that droplet-based scRNA-seq methods suffer from low mRNA capture efficiency, and the low abundance transcripts detected are not representative. However, cell lysis, mRNA capture, and reversed transcription can be efficiently carried out by improving the parameters of the microfluidic system, such as fluid speed and pressure [17–19]. In addition, hybridization of probes to RNA for sequencing (HyPR-seq), which can easily detect more than 100,000 cells in a single experiment, and achieve high sensitivity for individual transcripts in single cells and low-abundance transcripts [20]. However, multiple rounds of washes for probe hybridization and ligation, which result in some cell loss. Although the Smart-seq2 and Smart-seq3 schemes are more efficient in detecting genes than other scRNA-seq schemes, the sensitivity of detecting low-abundance genes was lower (0 < RPKM < 1, less than 2000 genes) [21, 22]. On the other hand, Wu et al. present to solve the problems of low capture efficiency, high noise, and high variability in single-cell sequencing by optimizing data analysis methods [23]. However, for large-scale consortium projects, experience has shown that neglecting benchmarking, standardization and quality control at the start can lead to major problems later on in the analysis of the results [24]. Therefore, we expect to optimize and improve the scRNA-seq scheme for the technical method to be suitable for ultralow RNA sequencing.

Based on the limitations of current scRNA-seq schemes, we aim to explore a library construction scheme for high sensitivity and low abundance gene detection ability, and it is suitable for subcellular or ultralow RNA sequencing (ulRNA-seq). We have optimized and improved from three aspects: Moloney murine leukemia virus (MMLV) reverse transcriptase (RT), template-switching oligos (TSO), terminal modification and template RNA structure. Using this protocol, we have sequence well-defined dilution series of total RNAs (5 pg, 2 pg, 1 pg, 0.5 pg), to comprehensively assess how mRNA capture efficiency, sensitivity, coverage uniformity, and detection of low abundance genes under different amounts of starting material, and it was verified in the single-cell micro-region obtained by glass hollow needle. Compared with the existing methods, this method can creatively apply an optimized and improved scRNA-seq to the precise analysis of spatial transcriptomes, subcellular, tissue biopsies, and rare samples such as circulating tumor cells and early developing embryonic cells, which improved the accuracy and reliability of single-cell sequencing results.

## Results

### Reverse transcription efficiency of different reverse transcriptases at the low amount of RNA input

Reverse transcriptase is the most important factor affecting the efficiency of reverse transcription. In this study, we compared the performance of five Moloney murine leukemia virus (MMLV) reverse transcriptase that has the necessary template-switching properties, each group had 3 technical replicates. cDNA yields are one of the most direct performance metrics of reverse transcriptase efficiency. The results showed that Template Switching showed higher cDNA yield to input 5 pg and 2 pg RNA (Supplementary Fig. S1A and B). However, at input amounts below 2 pg, Maxima H Minus reverse transcriptase showed higher cDNA yields, closely followed by SuperScript III (Supplementary Fig. S1C and D). In addition, we set up a quantitative reverse transcription (qRT-PCR) system, which can detect the efficiency of different reverse transcriptase based on the transcript abundance. Fig. S1E and F show the average Ct values of three genes measured by qPCR when using different reverse transcriptase, of which hypoxanthine phosphoribosyltransferase 1 (Hprt) is low-abundance expression in dopaminergic neurons, while 18S ribosomal (18S) and glyceraldehyde-3-phosphate dehydrogenase (GAPDH) are high-abundance expression. At input amounts 5 pg RNA, the Ct value of 18S and GAPDH was lower with Template Switching reverse transcriptase, while the Ct value of Hprt was lower with Maxima H Minus reverse transcriptase (Supplementary Fig. S1E). Therefore, this study shows that Maxima H Minus reverse transcriptase has a higher sensitivity for low expression genes. For the input amounts 0.5 pg RNA (except SMARTScribe reverse transcriptase), the results were similar. Using SMARTScribe reverse transcriptase, the Ct value of Hprt, 18S, and GAPDH were highest for input amounts 0.5 pg RNA (Supplementary Fig. S1F), indicating that the reverse transcription efficiency of SMARTScribe reverse transcriptase was the lowest.

### Maxima H minus reverse transcriptase improves the sensitivity of ulRNA-seq

**Fig. 1:**
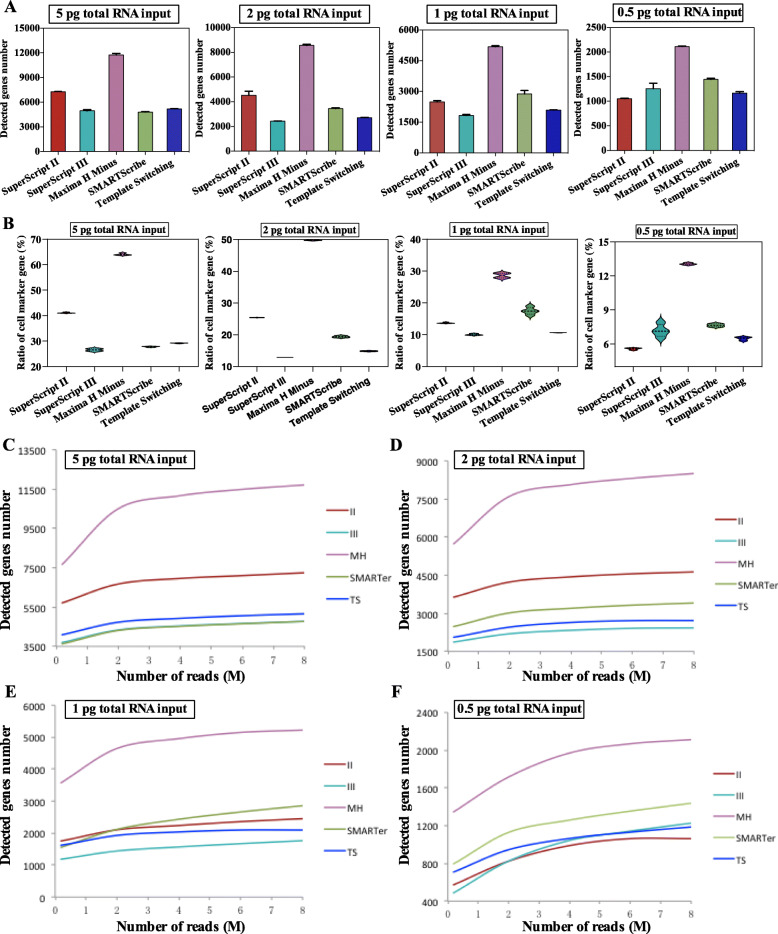
Gene detection sensitivity for the different reverse transcriptase library construction. **A** The number of genes detected per reverse transcriptase in different input amounts of RNA. **B** The ratio of the detected genes in the cell marker gene database of mice in different input amounts of RNA. **C**, **D**, **E**, **F** The collection curve showed the number of detected genes at different sequencing depths of different reverse transcriptase in the different input amounts of RNA

To improve the feasibility and sensitivity of single-cell or even subcellular library construction, the optimized library construction protocol was used to construct cDNA library for different input amounts of RNA. We evaluated the number of genes detected with 5 reverse transcriptases and 4 low-input RNAs. In this experiment, there were 3 technical replicates in each group, and a total of 60 libraries were constructed. The results showed the number of detected genes decreased with reduced input RNA by each reverse transcriptase, while the number of detected genes by Maxima H Minus reverse transcriptase were higher under different input amounts (Fig. 1A). Using Maxima H Minus reverse transcriptase, 11,754 genes were detected under 5 pg RNA input, which was less different from the number of genes detected in 1 ng RNA bulk sample (18,743 genes) (Fig. S2). We compared the detected genes to the identified cell marker gene database of mice, and calculated the ratio of the detected genes in the identified cell marker genes. The result showed that the mapping rate of Maxima H Minus reverse transcriptase under different RNA inputs was the highest, of which the mapping rate was 89.6% under 1 ng RNA input, 64.65% under 5 pg RNA input and 50.03% under 2 pg RNA input (Fig. 1B and Fig. S2). To compare the sensitivity of genes detected across protocols, an equal number of sequence depth was used per sample. The result showed that Maxima H Minus reverse transcriptase performed better compared with other reverse transcriptase (Fig. 1C-F). Overall, for the use of Maxima H- reverse transcriptase, the average number of genes detected at any depth of sequence is higher than any other reverse transcriptase. Besides, using Maxima H Minus and SMARTScribe reverse transcriptases, no obvious 3′- or 5′-end bias was observed in the transcripts detected under different input amounts of RNA (Supplementary Fig. S3A and B). Whereas SuperScript II, SuperScript III, and Template Switching reverse transcriptases show mild 5′-end bias (Supplementary Fig. S3A and B).

**Fig. 2:**
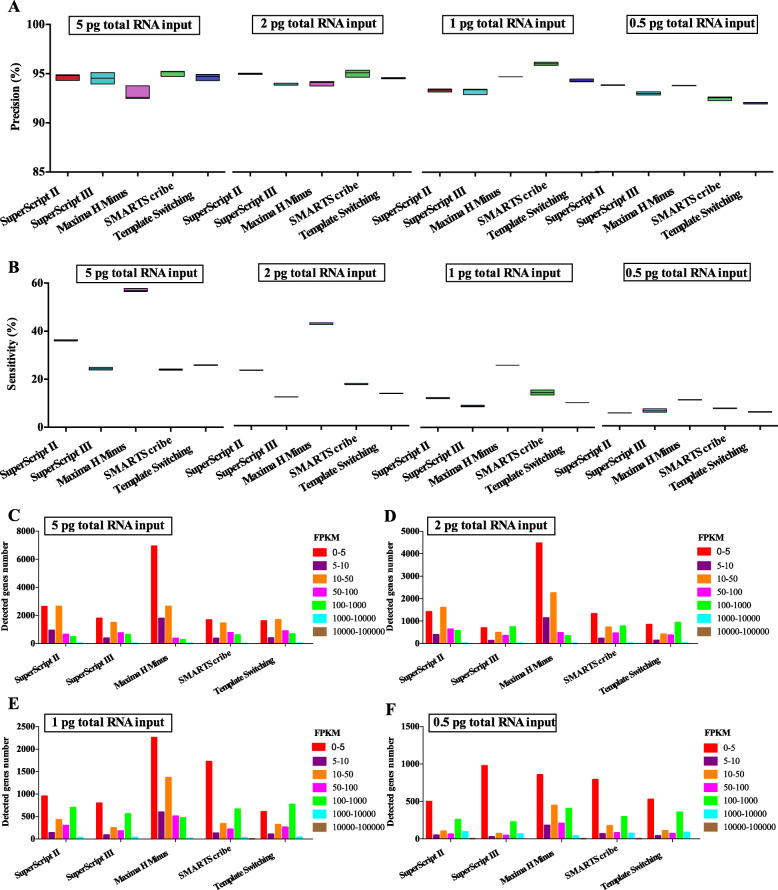
Precision and sensitivity of different reverse transcriptase library construction methods. **A** Precision for detecting genes at different reverse transcriptase and RNA input. **B** Sensitivity for detecting genes at different reverse transcriptase and RNA input. **C**, **D**, **E**, **F** Gene counts in different expression levels binned by standardized expression FPKM at different reverse transcriptase and RNA input. Different colors represent different FPKM values in Fig. **C**, **D**, **E**, and **F**

Maxima H Minus reverse transcriptase significantly increase sensitivity. Precision is considered to be the reproducibility of gene expression level estimation. Sensitivity assessment refers to the ratio of true positive genes detected at the same sequence depth. Using the 1 ng RNA input as a reference, we checked the precision (True positive/(True positive + False positive)) and sensitivity (True positive/(True positive + False negative)) at different input amounts of RNA in each reverse transcriptase. The result showed that as input decreased, precision remained robust among all five reverse transcriptases (Fig. 2A). Compare with other reverse transcriptase, Maxima H Minus reverse transcriptase had better sensitivity at different input amounts of RNA (Fig. 2B), and detect lower abundance genes (fragments per kilobase of transcripts per million (FPKM) at 0–5) (Fig. 2C-F). Therefore, we named the optimized library construction method ulRNA-seq.

**Fig. 3:**
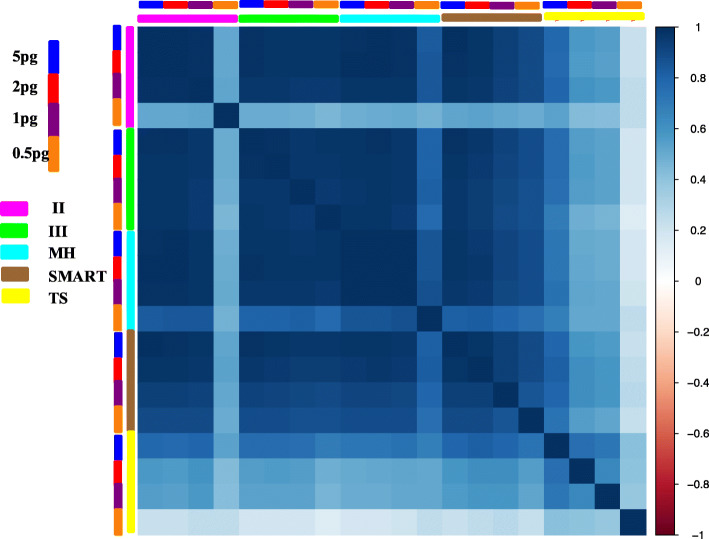
Heat map showing Pearson correlation of log2 transformed count values (Blue indicates high correlation and red indicates low correlation)

In addition, we examined the reproducibility of gene expression levels across different reverse transcriptase and RNA inputs. Overall, high reproducibility was observed across SuperScript II, SuperScript III, Maxima H Minus, and SMARTScribe (R^2^ ≥ 0.8), except the 0.5 pg RNA inputs from SuperScript II reverse transcriptase showing a lower level of concordance (R^2^ < 0.8) (Fig. 3). Template Switching reverse transcriptase showed a low correlation between other reverse transcriptase in different RNA input (Fig. 3).

### Terminal modification TSO improves the sensitivity and low abundance gene detection ability of ulRNA-seq

**Fig. 4:**
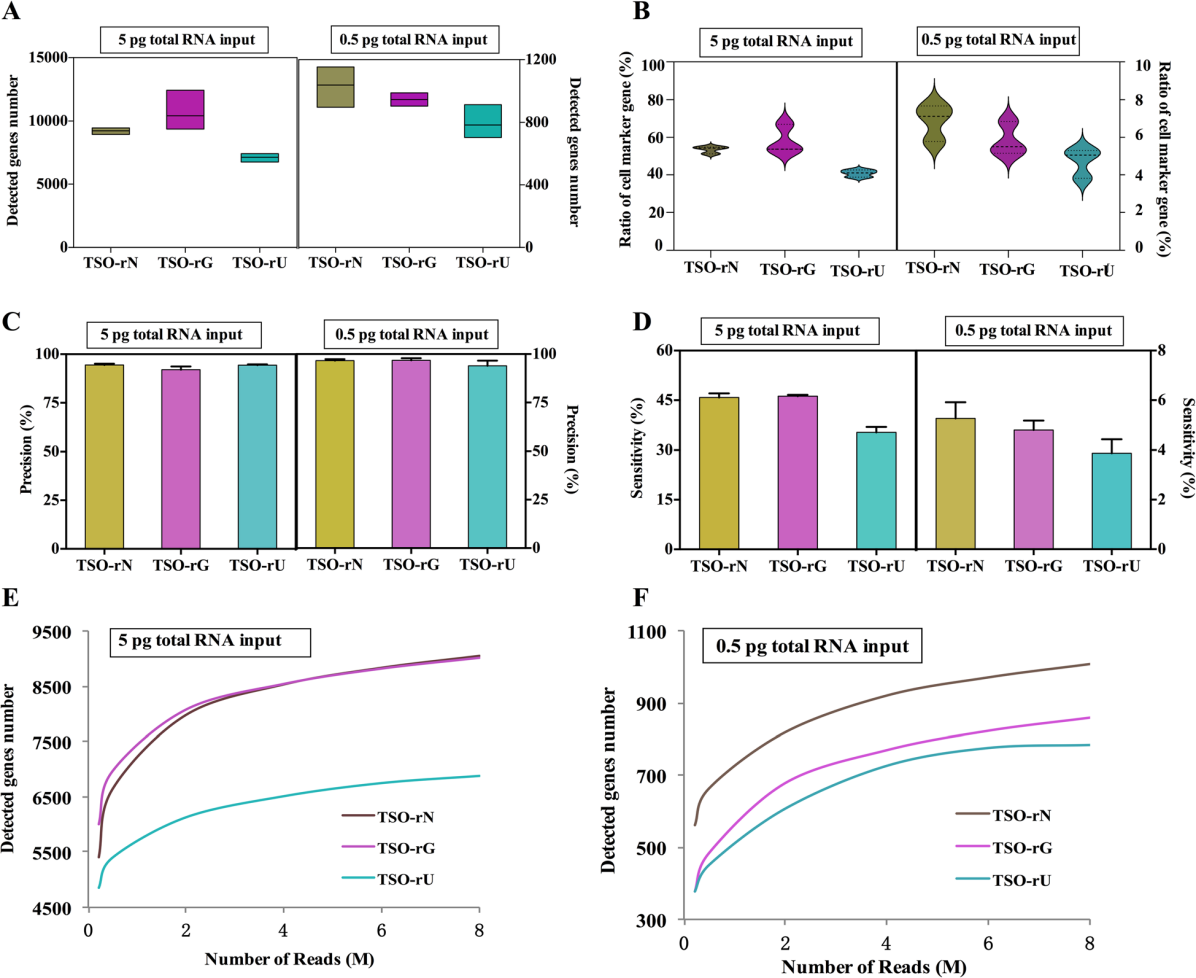
Sensitivity of different terminal modification TSO library construction methods. **A** The number of genes detected at 5 pg and 0.5 pg RNA inputs in different terminal modification TSO. **B** The ratio of the detected genes in the cell marker gene database of mice at 5 pg and 0.5 pg RNA inputs in different terminal modification TSO. **C** Precision for detecting genes in different terminal modification TSO. **D** Sensitivity for detecting genes in different terminal modification TSO. **E**, **F** The median number of genes detected per sample when downsampling total read counts to the indicated depths at 5 pg and 0.5 pg RNA inputs

Based on the ulRNA-seq protocol, the effects of different terminal modification TSO on library quality were compared. In this experiment, there were 3 technical replicates in each group, and a total of 18 libraries were constructed. We used multiple metrics to assess the quality of each library. The results are shown in Table S1. At 5 pg and 0.5 pg RNA input, the TSO-rN sequence detects a higher mapping rate, lower base sequence error rate, and more uniform GC content (Supplementary Table S1). We observed both more detected genes and cell marker genes with TSO-rN and TSO-rG sequence than TSO-rU sequences at input amounts 5 pg RNA (Fig. 4A, B). However, 0.5 pg RNA inputs, TSO-rN sequence detected the most genes and cell marker gene (Fig. 4A, B). Using the 1 ng RNA input as a reference, we checked the precision and sensitivity at 5 pg and 0.5 pg RNA inputs in different terminal modification TSO. The results showed that precision remained robust among all three libraries (Fig. 4C). The sensitivity of TSO-rU library was lower under 5 pg RNA inputs (Fig. 4D). However, sensitivity further dropped at 0.5 pg RNA inputs, with TSO-rN library showing higher sensitivity (Fig. 4D). The number of genes in each library identified (mean FPKM > 0) with different levels of sequence depth is shown in Fig. 4E, F. Under 5 pg RNA input, the number of genes identified using TSO-rN and TSO-rG sequences was significantly higher than that using TSO-rU with the increase of sequence depth (Fig. 4E) (*P* < 0.05). However, at 0.5 pg RNA inputs, TSO-rN detected more genes per sample at comparable sequence depth (Fig. 4F). These results showed that TSO-rN has high sensitivity for 0.5 pg RNA inputs, which indicates that the protocol is suitable for the construction of subcellular or ultralow input RNA libraries.

In addition, we performed principal component analysis (PCA) of all samples. In the MDS plot, the biological replicates clustered closely of the TSO-rN and TSO-rG samples under 5 pg RNA inputs (Supplementary Fig. S4A). Under 0.5 pg RNA input, the biological repetition of TSO-rN samples clustered closely (Supplementary Fig. S4B). Meanwhile, we also observed that the correlation between TSO-rN and TSO-rG samples was high (R^2^ > 0.98) (Supplementary Fig. S4C), and the technical reproducibility was nearly 1 (R^2^ > 0.98) (Supplementary Fig. S5A). However, the correlation between TSO-rU sample and the other two TSO sequences samples was low (R^2^ > 0.6) (Supplementary Fig. S4C), and the technical reproducibility was also low (R^2^ > 0.5) (in 0.5 pg RNA inputs) (Supplementary Fig. S5B).

**Fig. 5:**
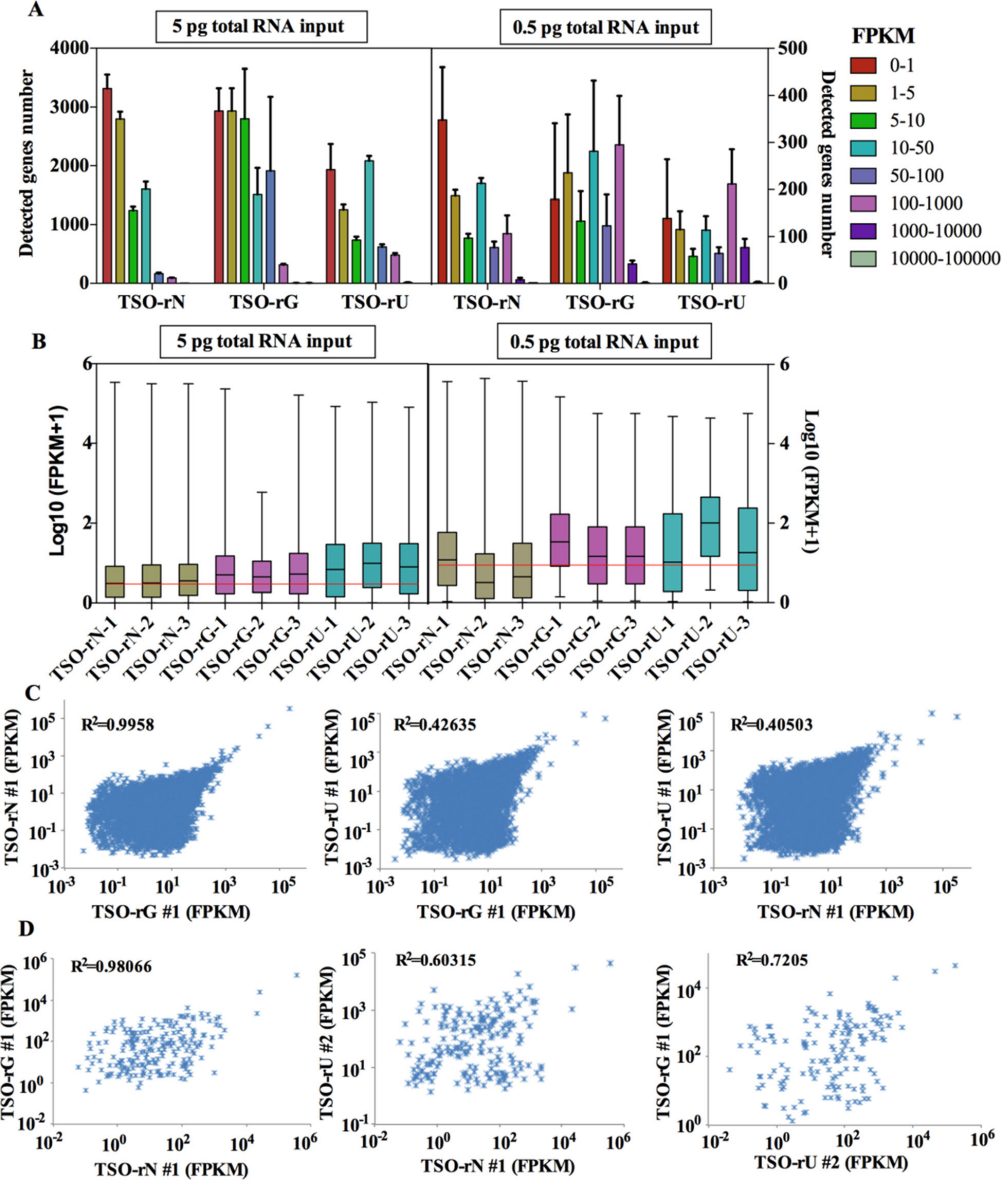
Effect of different terminal modification TSO on gene expression. **A** Gene counts in different expression levels binned by standardized expression FPKM at 5 pg and 0.5 pg RNA inputs in different terminal modification TSO. **B** Distribution of transcript quantification of all samples. **C** Scatter plots show the correlation between the different TSO terminal modifications at 5 pg RNA inputs. R^2^ indicates the coefficient of determination. **D** Scatter plots show the correlation between the different TSO terminal modifications at 0.5 pg RNA inputs. R^2^ indicates the coefficient of determination

We normalized the gene level expression data using FPKM to assess the agreement on the different libraries in terms of the number of genes captured and measurement of gene expression level. The gene number was detected by binning genes into 8 levels of expression of FPKM of 0–1, 1–5, 5–10, 10–50, 50–100, 100–1000, 1000–10,000, and 10,000–100,000. The results showed that most genes were detected in FPKM at 0–1, indicating that more genes with low expression could be detected using TSO-rN sequence at 5 pg and 0.5 pg RNA inputs (Fig. 5A, B). The gene expression correlation results of different TSO sequences are shown in Fig. 5c, d. The correlation between gene expression of TSO-rN and TSO-rG samples is very high, but the correlation between TSO-rU samples is relatively low (Fig. 5C, D). In addition, we compared the effects of different TSO sequences on the alternative splicing (AS) events (exon skipping (ES), alternative donor (AD), alternative adaptor (AA), mutually exclusive exon (MXE), intron retention (IR)). The results showed that there was few differential expression alternative splicing (DEAS) events between different groups, of which the difference ES events were about 200, indicating that the different TSO sequences had no effect on the AS analysis (Supplementary Fig. S6).

### The effect of different RNA structures on gene detection and RNA quantification

**Fig. 6:**
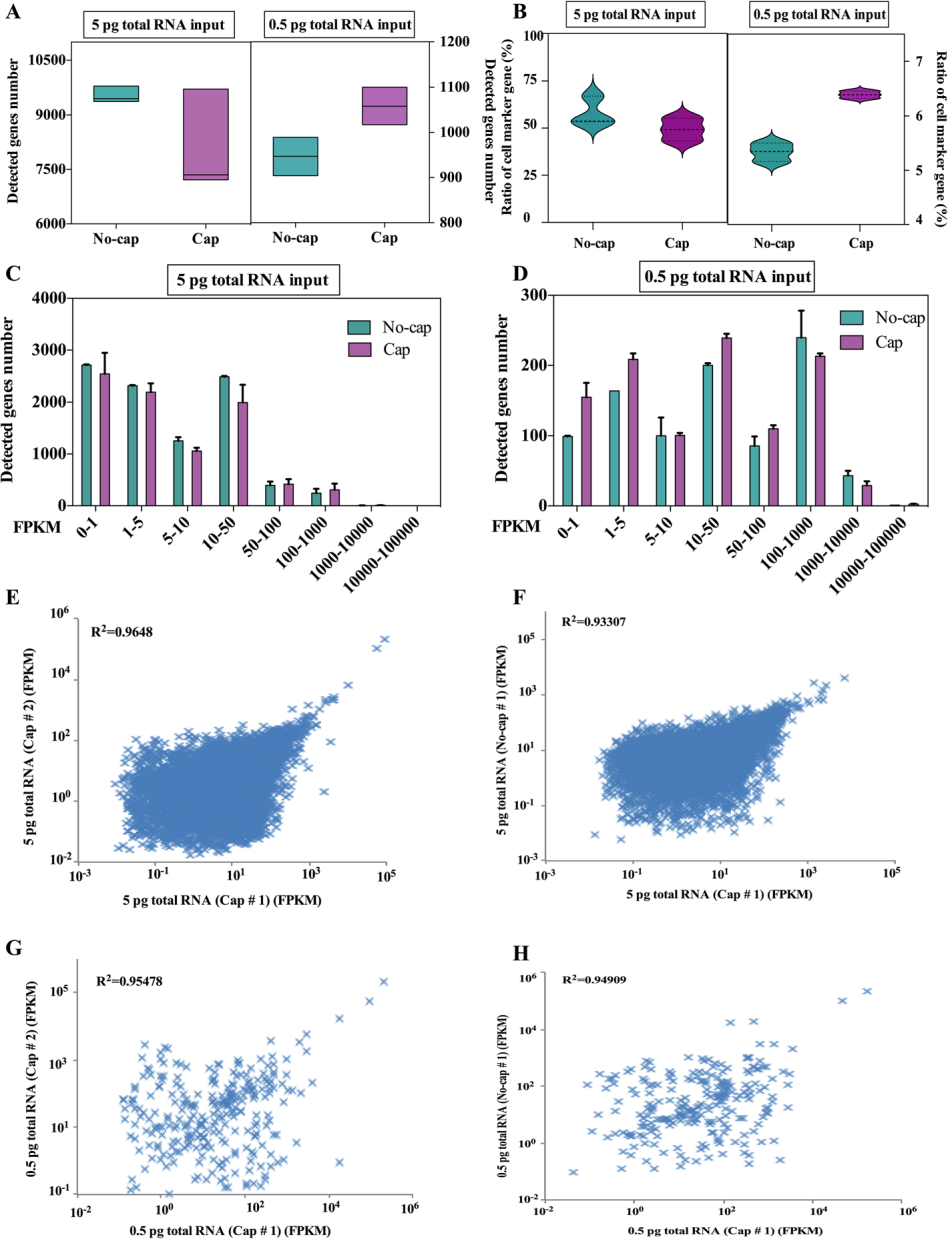
The effect of RNA with different template structures on gene detection. **A** The number of genes detected in the different structure of mRNA templates. **B** The ratio of the detected genes in the cell marker gene database of mice at the different structures of mRNA templates. **C**, **D** Number of genes detected in different expression levels binned by standardized expression FPKM at the different structures of mRNA templates. **E**, **F** Scatter plots show the correlation between different replicates and mRNA structure for 5 pg RNA inputs. **G**, **H** Scatter plots show the correlation between different replicates and mRNA structure for 0.5 pg RNA inputs

In this study, we sequence m7G-capped RNA and uncapped RNA templates as a way to compare the performance of scRNA-seq. In this experiment, there were 3 technical replicates in each group, and a total of 12 libraries were constructed. Using the ulRNA-seq protocol, we detected more genes and cell marker genes in 5 pg uncapped RNA templates (detected 9413 genes and 53.6% cell marker genes), but there was no significant difference compared with m7G-capped RNA (Fig. 6A, B). However, m7G-capped RNA templates were detected more genes and cell marker genes than uncapped RNA in 0.5 pg RNA input (Fig. 6A, B) (*P* < 0.05). In addition, we also examined the influence of the two libraries on the number of genes in the range of FPKM values. The figure showed that the template RNA structures didn’t effect on gene expression at 5 pg RNA input (Fig. 6C). However, at 0.5 pg RNA inputs, the number of genes detected by m7G-capped RNA template was significantly higher than that of uncapped RNA in FPKM 0–1, 1–5, 10–50, and 100–1000 groups (Fig. 6D). We examined the reproducibility of gene expression levels across protocols and technical replicates. The result showed that the correlation of protocols and technical replicates showed a high correlation (R^2^ > 0.95 for m7G-capped RNA and uncapped RNA sequencing) (Fig. 6E-H).

**Fig. 7:**
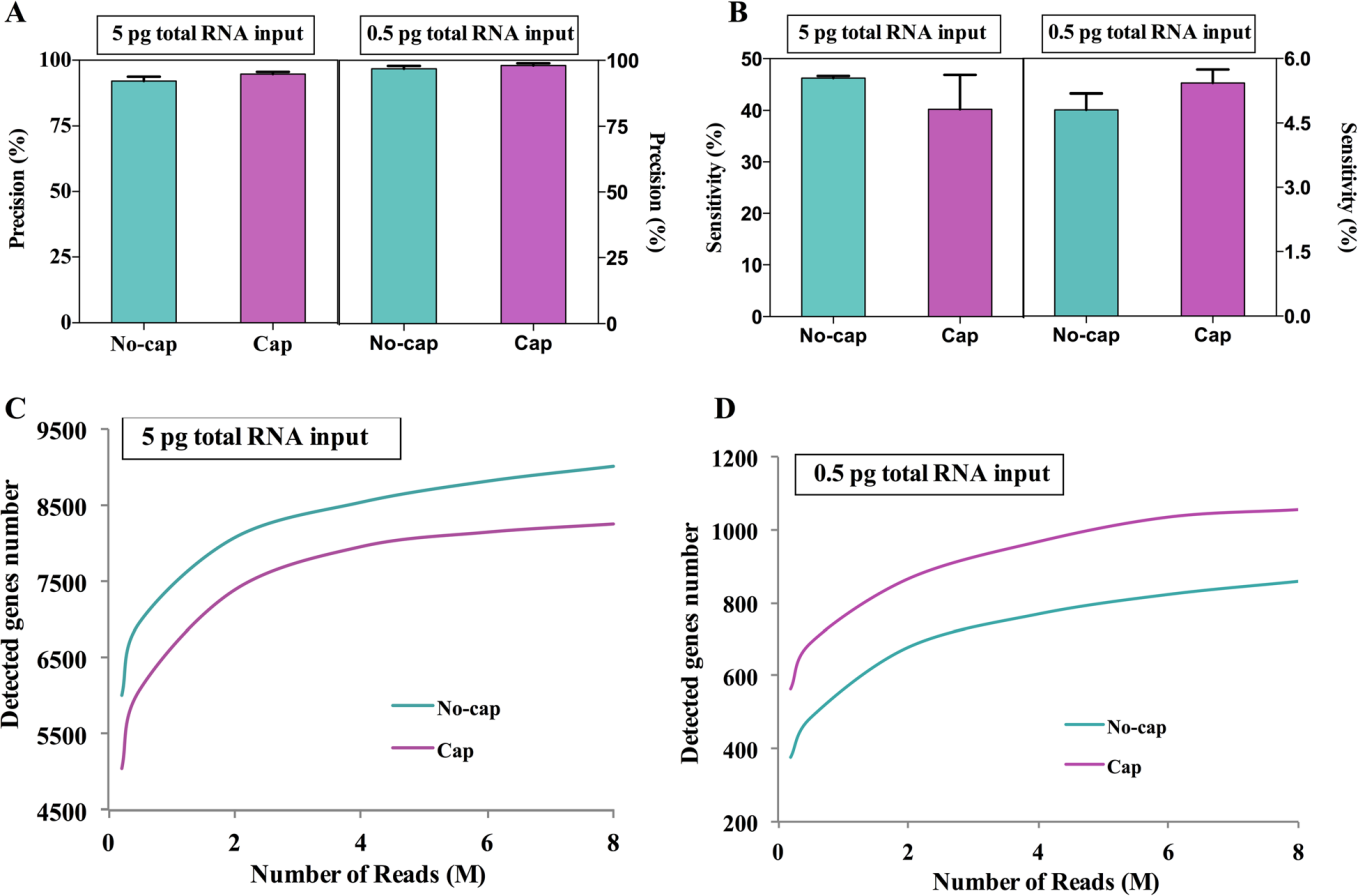
Effect of RNA with different template structures on the accuracy and sensitivity of sequencing. **A** Precision for detecting genes at the different structures of mRNA templates. **B** Sensitivity for detecting genes at the different structures of mRNA templates. **C**, **D** The median number of genes detected per sample when downsampling total read counts to the indicated depths at 5 pg and 0.5 pg RNA inputs

In addition, the precision and sensitivity are affected by the template RNA structure. Using 1 ng RNA input as a reference, the precision and sensitivity of sequence different internal RNA sequences were analyzed. The sequence precision of different input amounts to m7G-capped RNA and uncapped RNA templates were about 92% ~ 98%, and there was no difference between the two RNA templates (Fig. 7A). However, using 0.5 pg total RNA inputs, m7G-capped RNA showed higher sensitivity (Fig. 7B). Next, we analyzed the impact of sequencing depth on the detection of genes. From a cost perspective, researchers can control the sequencing depth to fit their budgets and needs, which is especially important for the scRNA-seq experiment. We found that sensitivity saturated at 5 million reads per sample (Fig. 7C, D). Using 5 pg total RNA inputs, uncapped RNA template was more sensitive, while using 0.5 pg total RNA inputs, m7G-capped RNA was more sensitive (Fig. 7C, D). Coverage analysis of gene showed that m7G capped RNA showed more uniform 5′ to 3′ gene coverages, while uncapped RNA template showed 3′ biases under 0.5 pg RNA inputs (Supplementary Fig. S7A, B). In addition, we compared five important AS events of different template RNA structure data sets. The results showed that there were few DEAS, of which only 255 were detected by DESE, which may be caused by the difference caused by sequencing (Supplementary Fig. S8). This reveals that the template RNA structure has no significant effect on the AS events of the sample.

### Verify the sensitivity of ulRNA-seq protocol in single-cell

**Fig. 8:**
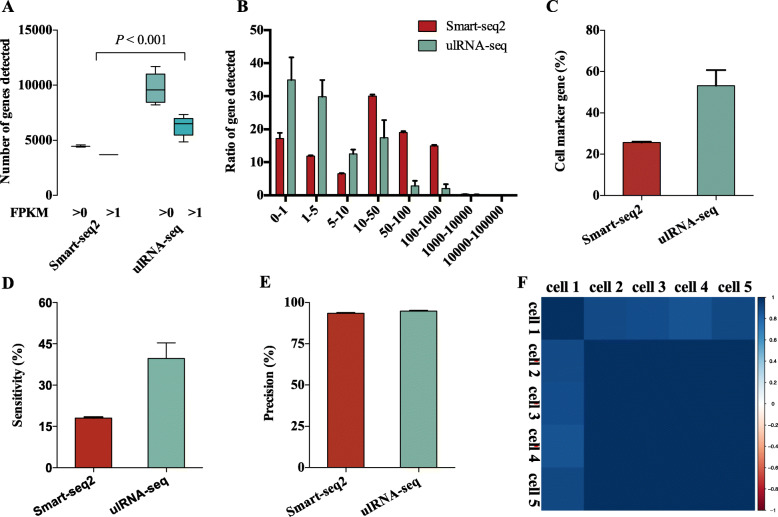
Validation of ulRNA-seq protocol using single-cell micro-region. **A** The number of genes detected Smart-seq2 and ulRNA-seq protocol. **B** The number of genes detected in different expression levels binned by standardized expression FPKM in single-cell. **C** The ratio of the detected genes in the cell marker gene database of mice. **D** Sensitivity for detecting genes in single-cell. **E** Precision for detecting genes in single-cell. **F** Heatmap showing person correlation between the five replicates

Finally, we applied Smart-seq2 and ulRNA-seq protocol to single cells micro-region. By using a fine glass hollow needle with a diameter of 28 μm obtained single-cell samples by punching tissue slices from the same slice to verify the sensitivity and feasibility of the optimized protocol. In this experiment, Smart-seq2 data and ulRNA-seq data have 3 and 5 technical replicates respectively, and a total of 8 libraries were constructed. Using the ulRNA-seq protocol can detect more total genes (9725 genes) and low abundance genes (Fig. 8A, B). And 53.2% of the genes in the cell marker database were detected in single cells (Fig. 8C). In addition, compared with the Smart-seq2 protocol, ulRNA-seq has higher sensitivity in single-cell library construction (Fig. 8D), but there is no difference in precision between the two protocols (Fig. 8E). The high reproducibility between different samples reveals the high stability of the ulRNA-seq protocol (Fig. 8F).

## Discussion

The rapid development of single-cell genomics has transformed our understanding of biological systems. However, the limitations of single-cell library construction, such as dropout probability and low mRNA capture efficiency, limit the sensitivity of gene detection and the accuracy of cell subtype analysis, thus affecting the reliability of downstream analysis results. Different single-cell sequencing methods have different dropout probability, among which MARS-seq had the highest median dropout probability (74%) and Smart-seq2 had the lowest (26%) [25], dropout event will lead to many low expression or medium expression genes that can’t be effectively detected. Single-cell sequencing technology based on the microfluidic system can effectively improve mRNA capture efficiency through adjusting the controllable valve and pump, but the total number of genes detected by this scheme is less than 6000 [17]. However, the use of poly (A) tagging strategy improved the efficiency of conversion to a mRNA molecule to amplified cDNA, and used unique molecular identifiers (UMI) tags for each cell can increase the detection of low expression genes [19]. Although the sequencing technology based on probe hybridization can improve the detection number of cells and transcript detection sensitivity, probe hybridization requires multiple cleaning, resulting in the loss of some cells [20]. Therefore, different single-cell technologies have different limitations. We expect to optimize and improve the single-cell library construction method to improve the sensitivity of sequencing results. In addition, the current research of subcellular RNAs is still limited. High throughput sequencing of subcellular RNA can be used to reveal the identity, abundance, and subcellular distribution of transcripts, thus providing insights into RNA processing and maturation [26]. This will provide an important resource to explain the complex subcellular structure, cell dysfunction, and pathophysiology of human diseases. Here, the total RNA of different gradients is used to simulate the level of single-cell or subcellular. We systematically analyze the effects of five reverse transcriptases, three TSO terminal modifications, and two template RNA structures on single-cell data, to provide a highly sensitive library construction scheme for subcellular and spatial transcriptomics related research. As far as we know, this is the first time to compare the effects of TSO terminal modification and RNA template structure on the sensitivity of scRNA-seq. We expect to generate high sensitivity transcriptomes from single cells or subcellular according to the ulRNA-seq protocol, to provide high-resolution inspections of basic processes such as cell differentiation and carcinogenesis.

For single-cell and low-input RNA library preparation, template switching-based RNA-seq is increasingly gaining popularity. However, the efficiency of reverse transcription and template switching can affect single-cell mRNA capture, thus reducing the sensitivity of single-cell sequencing results. Among them, reverse transcriptase is the most direct factor affecting the efficiency of reverse transcription [27]. In this study, we used cDNA yield and low-abundance gene quantification to characterize the reverse transcription efficiency, and the results showed that the reverse transcription efficiency is variable with different template input, which is consistent with the results of other studies [28]. In addition, we also found that Maxima H Minus reverse transcriptase significantly improved the efficiency of gene detection, with higher accuracy and sensitivity, so Maxima H Minus reverse transcriptase is preferable for single-cell application. Hagemann-Jensen et al.’s studies also showed that Maxima H Minus reverse transcriptase could detect more genes than SuperScript II reverse transcriptase in scRNA-seq [22]. The possible reason is that Maxima H Minus reverse transcriptase is engineered to have minimal RNase H activity to improve their processivity, robustness, and synthesis rate. In addition, Maxima H Minus reverse transcriptase is thermostable, which can be used at higher reaction temperatures within the protocols. Previous reports have shown that destabilization of the secondary RNA structures at increased temperature leads to more frequent primer hybridization and stable reverse transcription, which may be the main reason for the high efficiency of Maxima H Minus reverse transcription [29, 30]. Our findings are in accordance with Bagnoli et al. report, indicating that the selection of reverse transcriptase is an important factor affecting the sensitivity of single-cell sequencing [31]. It is also worth noting that the sequencing depth of transcripts is not only related to sequencing cost, but also closely related to the number of genes detected and the accuracy of gene expression. In this study, Maxima H Minus reverse transcriptase detected more low abundance genes at the same sequencing depth, which effectively reduced the risk of missed detection of low-abundance mutations in clinical samples and found more clinical treatment opportunities. In addition, using Maxima H Minus reverse transcriptase to detect more cell marker genes in a single cell is helpful to the accurate identification of cell types, to better understand the biological functions and characteristics of cells. Therefore, we proved that Maxima H Minus reverse transcriptase has the best mRNA capture efficiency and sensitivity regardless of the amount of input RNA.

Moreover, it has been reported that TSO terminal modification or mRNA templates may lead to inefficient amplification and therefore to affect the efficiency of mRNA capture [32–34]. Due to the base preference of the terminal transferase activity of Maxima H Minus reverse transcriptase, we compared three TSO terminal modifications. The results showed that the use of randomized TSO, such as TSO-rNrG+G, showed higher accuracy and sensitivity for 0.5 pg total RNA, improved the efficiency of mRNA capture, and detected more low abundance genes. Pawel et al. showed that with increasing distance from the end of the transcript, the preference of reverse transcriptase for cytosine decreased, so setting a degenerate base at the third ribose base at the 3 ´-most positions could capture more cDNA molecules [32]. In addition, the results also show that RNA cap structures have more uniform coverage, higher sensitivity and reproducibility for 0.5 pg total RNA, and increase the detection efficiency of low abundance genes, so they are more suitable for single-cell or subcellular samples. Wulf et al.’s studies have shown that for uncapped RNAs, some transcripts may be over-represented in sequencing reads, which limits the accuracy of small RNA and highly degraded RNA sequencing results [33]. However, m7G-capped RNA has higher template switching efficiency and smaller sequencing bias. The possible reason is that the cap structure somehow stalls reverse transcription, allowing more time for TSO to interact and allow template switching to occur, thus improving the efficiency of mRNA capture [33]. Vahrenkamp et al.’s studies have also shown that adding a cap structure to FFPE-derived RNA can significantly improve the quality of the library and accurately quantify the transcript [34]. Therefore, optimization of the relevant parameters in the library reaction, such as TSO and mRNA template structure, can further improve the template-switching efficiency and mRNA capture efficiency, thereby improving the sensitivity and accuracy of the ultra-low trace RNA sequencing results, and constructing a truly unbiased scRNA-seq platform. However, this protocol may not be suitable for low-quality samples, as oligo-dT used in reverse transcription will cause gene coverage bias. Therefore, random primers can obtain more representative and comprehensive transcriptome information for degraded samples. In addition, we believe that ulRNA-seq can be applied to animals, plants, and microorganisms. Although there are great differences in RNA content and GC content between different species, the structure of RNA is similar. mRNA capture efficiency and cDNA library yield can be increased by changing reverse transcriptase and increasing the PCR cycle. In addition, the library construction method uses oligo-dT anchored primers for RNA capture, so it will not cause base imbalance in sequencing results due to GC content deviation. Previous studies have shown that ultra-low input RNA-seq analysis based on Smart-seq2 has been applied in animal liver, lymphoid cell, fungi, and single-nematode [35–38].

At present, our results show that the ulRNA-seq protocol has higher sensitivity at 0.5 pg total RNA input. Therefore, this protocol may be applied to ultralow input RNA samples, subcellular, or high-resolution spatial transcriptome-related research. When we obtained a single cell with spatial position, it may be not a complete cell. The preparation of tissue sections leads to the loss or degradation of RNA in a single cell, so that ultralow RNA sequencing can be performed at the RNA input level lower than single-cell levels. Liu et al. developed the DBiT-seq technology and detected 2068 genes in approximately 4 pg of total RNA [39]. We believe that the ulRNA-seq protocol may achieve higher spatial resolution, and not only can increase the number of genes detected per data point, but also increase the identification of low-abundance expressed genes. To show the high sensitivity of the ulRNA-seq protocol, we applied this protocol to single-cell microregion samples and systematically compared its performance. The results showed that 9725 genes and more low abundance genes were detected in mouse brain single cells. Yamazaki et al. only detected 8598 genes in mouse brain single cells using Smart-seq2 [38]. However, low coverage may lead to poor cell-type identification, which may result in some rare cell types being undetectable. Therefore, it is suggested that the ulRNA-seq library protocol has higher mRNA capture ability and low abundance gene detection ability. However, our validation experiment also has limitations. Since the sample obtained by glass hollow needle is not a single-cell sample, the results of this study may be different from those of high-throughput single-cell isolation.

## Conclusion

In summary, we presented a template-switching based library preparation method in the study, which obtained higher sensitivity, accuracy, and gene detection ability in ultralow input RNA sequencing. It is concluded that Maxima H Minus reverse transcriptase, rN modified TSO and 5′-capped RNA templates contributed main to the enhanced ability of low abundance gene detection and ultralow RNA input in this method. More gene numbers and cell markers were identified, and more uniform coverage was obtained in single-cell sequencing. It is suggested that ulRNA-seq will help the further development of single-cell and subcellular studies.

## Methods

### Animals and sample collection

One male C57Bl/6 J mouse (8 weeks old) was purchased from Shanghai Southern Model Biotechnology Co., Ltd. It was anesthetized with tribromoethanol (500 mg/kg) (Sigma, Saint Louis, USA), and then was killed by cervical dislocation. This study was reviewed and approved by the Ethics Committee of Zhongda Hospital Southeast University. The brain was dissected from the skull, and then the brain sample was washed with 0.9% pre-cooled saline. The brain samples were immediately treated to isolate the RNA.

### Total RNA isolation and experimental design

Total RNA was extracted from brain tissue according to the method of Chomczynski and Sacchi [40]. The RNA integrity number (RIN) value was determined using the Agilent 4200 Bioanalyzer and high sensitivity RNA screen tape kit according to the manufacturer’s instructions. We selected samples with RIN value greater than 8.5 for dilution. RNA samples for the RT assays were prepared (serial dilution from the same RNA pool) in aliquots of 0.5, 1, 2, and 5 pg total brain RNA. Five reverse transcriptases, Maxima H- (Thermo Fisher), SMARTScribe (Clontech), Superscript II (Thermo Fisher), Superscript III (Thermo Fisher), and Template Switching RT Enzyme Mix (New England Biolabs), were evaluated for their ability to template switch and efficiency of reverse transcription. Several dilutions ranging from 0.5, 1, 2, and 5 pg of total RNA were used as input for the RT reactions. In addition, we use 1 ng of input RNA sample (bulk sample) as a control. In this study, a total of 116 samples were analyzed.

### Primer sequences

The oligonucleotide sequences are listed in Table 1. Oligo-dT used for the synthesis of the first RNA strand is an anchored primer. We designed three different TSO terminal modifications. Then, the cDNA library was constructed with different TSO sequences.

**Table 1:** Oligonucleotide sequences

Oligonucleotide	Sequences
**Oligo-dT**	5′-AAGCAGTGGTATCAACGCAGAGTACT_30_VN-3′
**TSO-rN**	5′ Biotin-AAGCAGTGGTATCAACGCAGAGTACATrNrG+G-3′
**TSO-rG**	5′ Biotin -AAGCAGTGGTATCAACGCAGAGTACATrGrG+G-3′
**TSO-rU**	5′ Biotin -AAGCAGTGGTATCAACGCAGAGTACATrGrU+G-3′

### Preparation of capped RNA templates

Whether the extracted RNA is complete or not, we add capped structure to the extracted total RNA. Capping RNA templates were performed using the Vaccinia Capping System (catalog number M2080). Briefly, 5 pg or 0.5 pg RNA, 1 × Capping Buffer, 0.5 mM GTP, 0.1 mM SAM, 2.5 units of Vaccinia Capping Enzyme were incubated for 30 min at 37 °C. Then, the cDNA library was constructed with capped RNA templates.

### Single-cell library preparation and sequencing

First, the 2 μl capped RNA templates, 0.5 μl 10uM oligo-dT primer, 1 μl 10 mM dNTP mix, and 0.25 μl 40 U μl^− 1^ RNAse inhibitor were at 72 °C for 3 min for denaturation, and immediately placed on ice afterward. Next, 2.5 μl 5 X first-strand buffer, 1 μl 10 μM TSO primer, 2 μl 5 M betaine, 1 μl 25 mM MgCl_2_, 0.5 μl 0.1 M DTT, 0.25 μl 40 U μl^− 1^ RNAse inhibitor, and 1 μl 200 U μl^− 1^ reverse transcriptase were added to each sample. Different reverse transcriptases have different template switching conditions. The reaction program of Superscript II reverse transcriptase is 42 °C, 90 min, then 10 cycles (50 °C, 2 min; 42 °C, 2 min), and finally 70 °C, 15 min. The reaction program of Superscript III reverse transcriptase is 50 °C, 60 min, and 70 °C 15 min. The reaction program of Maxima H reverse transcriptase is 42 °C, 90 min, then 10 cycles (50 °C, 2 min; 42 °C, 2 min), and finally 85 °C, 5 min. The reaction program of SMARTScribe reverse transcriptase is 42 °C, 90 min, and 70 °C, 10 min. The reaction program of Template Switching RT Enzyme Mix is 42 °C, 90 min, and 85 °C, 5 min. PCR pre-amplification was performed directly after reverse transcription by adding PCR mix, containing 12.5 μl 2 X KAPA HiFi HotStart Ready Mix and 0.5 μl 5 μM PCR primer. The number of PCR cycles depends on the input amount of RNA. We typically use 20 cycles for than 100 pg RNA input. The number of cycles can be increased to 25 cycles for 1 pg ~ 50 pg RNA input. We use 30 cycles for less 1 pg RNA input. PCR was cycled as follows: 3 min at 98 °C for initial denaturation, 25 cycles of 20 s at 98 °C, 15 s at 67 °C and 6 min at 72 °C. Final elongation was performed for 5 min at 72 °C. Then, we measured the cDNA concentration using the Qubit dsDNA Assay Kit (Thermo Fisher). Finally, 1 ng of cDNA was then used for the tagmentation reaction carried out with One-step DNA Lib Prep Kit for Illumina (ABclonal), with the addition of Tagment DNA Buffer and Tagment DNA Enzyme, in a final volume of 50 μl. The tagmentation reaction was incubated at 55 °C for 5 min and then purified with Ampure XP beads. After tagmentation, the Tn5 transposon DNA will add adaptor adapters at both ends of the RNA/DNA hybrid strand for subsequent PCR amplification for the library building. The reaction program is 72 °C, 3 min, 98 °C, 45 s, then 13 cycles (98 °C, 15 s; 60 °C, 30 s; 72 °C, 3 min), and finally 72 °C, 5 min. The Agilent 2100 High Sensitivity DNA Assay Kit was used to detect the distribution of amplified product cDNA fragments. According to the detection results, the quality of the amplified product cDNA was determined, and the subsequent cDNA library was sequenced on the Illumina HiSeq X10 PE150 platform (Illumina, USA).

### Data analysis

Firstly, the raw data were filtered to generate clean data, and FastQC software (v0.11.4) was used for the quality control (QC) of the sequencing data. Then, clean data were aligned to the mouse reference sequences by Hisat2 software using default parameters. The expression levels of each transcript were normalized by quantifying FPKM.

### Quantitative reverse transcription (qRT-PCR) analysis

To verify the efficiency of different reverse transcriptases, quantitative real-time PCR (qPCR) was used to analyze GAPDH, 18S [41], and Hprt1 [41] (Table 2). The reaction program was set as follows: for 30 s at 95 °C; 40 PCR cycles (95 °C, 5 s; 60 °C, 34 s (fluorescence collection)). The relative expression of target genes was calculated by the 2 ^-△△CT^ method.

**Table 2:** Nucleotide sequences of the primers were used to assay gene expression by RT-qPCR

Genes	Sequences
**GAPDH**	F: 5′-CGTCCCGTAGACAAAATGGT-3′
	R: 5′-TTGATGGCAACAATCTCCAC-3′
**18S**	F: 5′-AAACGGCTACCACATCCAAG-3′
	R: 5′-CAATTACAGGGCCTCGAAAG-3′
**Hprt1**	F: 5′-CAAACTTTGCTTTCCCTGGT-3’
	R: 5′-CTGGCCTGTATCCAACACTTC-3’

Note: F, forward primer; R, reversed primer

## Supplementary Information

**Additional file 1: Table S1.** Summary of ulRNA-seq.

**Additional file 2: Figs. S1.** Compared the efficiency of different reverse transcriptase. **Figs. S2.** The number of genes and cell marker genes detected at 1 ng RNA inputs. **Figs. S3.** Gene body coverage averaged over different input amounts of RNA sequenced with the different reverse transcriptase library construction. **Figs. S4.** Principal component analysis and correlation analysis of normalized gene expression values for all samples RNA-Seq datasets analyzed. **Figs. S5.** Correlation analysis for all samples RNA-Seq datasets. **Figs. S6.** Comparison of differentially expressed alternative splicing (DEAS) under the different terminal modification TSO for 5 pg RNA inputs. **Figs. S7.** Gene body coverage averaged over the different structure of mRNA templates. **Figs. S8.** Comparison of differentially expressed alternative splicing (DEAS) under the different structures of mRNA templates for 5 pg RNA inputs.

## Data Availability

The RNA-seq datasets are available at NCBI project PRJNA742432 (https://dataview.ncbi.nlm.nih.gov/object/PRJNA742432) and PRJNA741817 (https://dataview.ncbi.nlm.nih.gov/object/PRJNA741817).

## Abbreviations

scRNA-seq: Single-cell RNA sequencing
ulRNA-seq: Ultralow input RNA sequencing
TSO: Template-switching oligos
HyPR-seq: Hybridization of probes to RNA for sequencing
MMLV: Moloney murine leukemia virus
RT: Reverse transcriptase
RIN: RNA integrity number
QC: Quality control
FPKM: Fragments per kilobase of transcripts per million
qRT-PCR: Quantitative reverse transcription
GAPDH: Glyceraldehyde-3-phosphate dehydrogenase
18S: 18S ribosomal
Hprt1: Hypoxanthine phosphoribosyltransferase 1
UMI: Unique molecular identifiers
