# Correction to: Optimization of library preparation based on SMART for ultralow RNA-seq in mice brain tissues

**DOI:** 10.1186/s12864-022-08322-0

**Published:** 2022-02-08

**Authors:** Erteng Jia, Huajuan Shi, Ying Wang, Ying Zhou, Zhiyu Liu, Min Pan, Yunfei Bai, Xiangwei Zhao, Qinyu Ge

**Affiliations:** 1 State Key Laboratory of Bioelectronics, School of Biological Science & Medical Engineering, Southeast University, Nanjing, 210096 China; 2 School of Medicine, Southeast University, Nanjing, 210097 China

**Correction to: BMC Genomics 22, 809 (2021)**

**https://doi.org/10.1186/s12864-021-08132-w**

Following publication of the original article [1], it was reported that there were errors in the x- and y-axes of Figs. 4, 6 and 7.

**Fig. 4:**
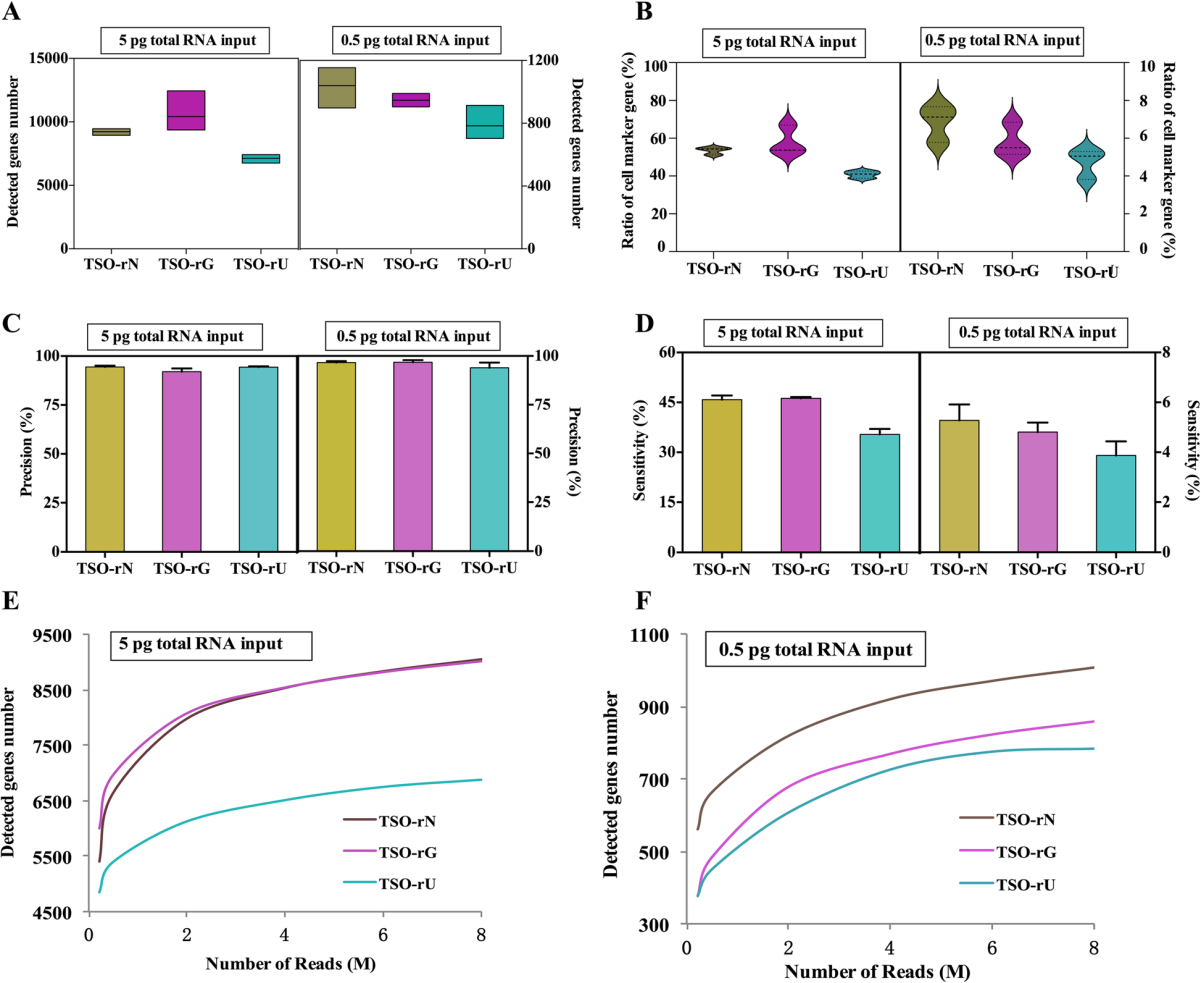
Sensitivity of different terminal modification TSO library construction methods. **A** The number of genes detected at 5 pg and 0.5 pg RNA inputs in different terminal modification TSO. **B** The ratio of the detected genes in the cell marker gene database of mice at 5 pg and 0.5 pg RNA inputs in different terminal modification TSO. **C** Precision for detecting genes in different terminal modification TSO. **D** Sensitivity for detecting genes in different terminal modification TSO. **E, F** The median number of genes detected per sample when downsampling total read counts to the indicated depths at 5 pg and 0.5 pg RNA inputs

**Fig. 6:**
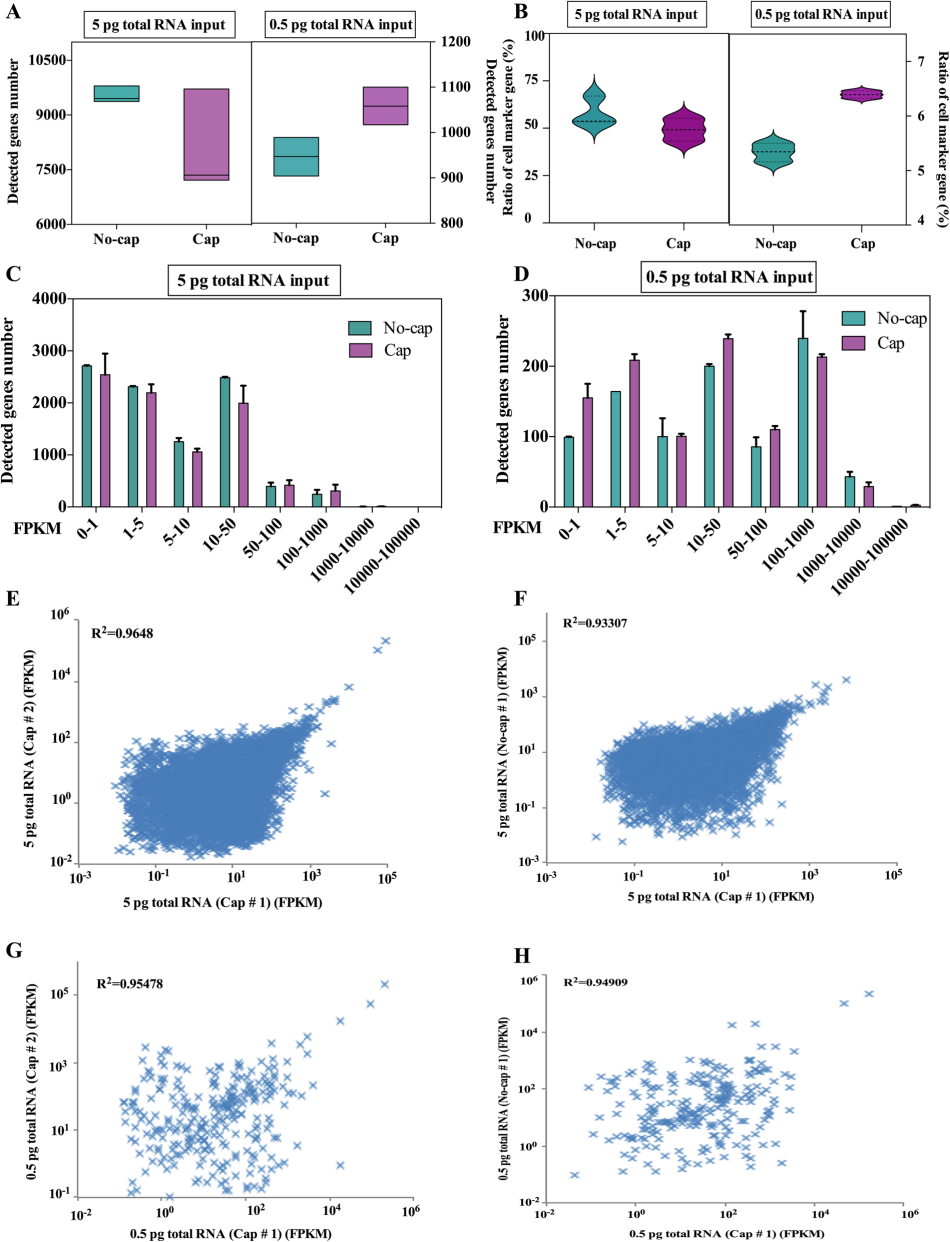
The effect of RNA with different template structures on gene detection. **A** The number of genes detected in the different structure of mRNA templates. **B** The ratio of the detected genes in the cell marker gene database of mice at the different structures of mRNA templates. **C, D** Number of genes detected in different expression levels binned by standardized expression FPKM at the different structures of mRNA templates. **E, F** Scatter plots show the correlation between different replicates and mRNA structure for 5 pg RNA inputs. **G, H** Scatter plots show the correlation between different replicates and mRNA structure for 0.5 pg RNA inputs

**Fig. 7:**
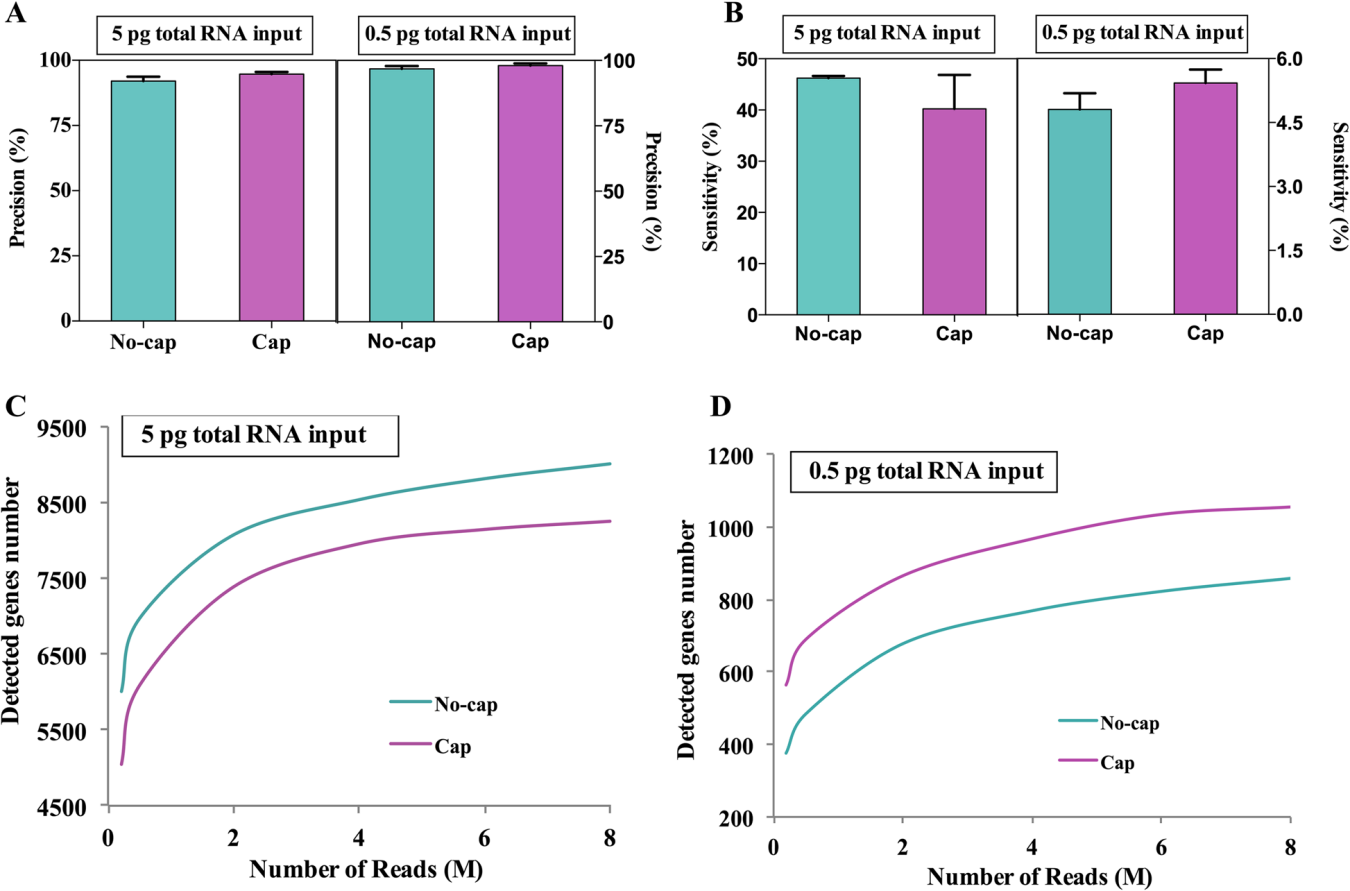
Effect of RNA with different template structures on the accuracy and sensitivity of sequencing. **A** Precision for detecting genes at the different structures of mRNA templates. **B** Sensitivity for detecting genes at the different structures of mRNA templates. **C, D** The median number of genes detected per sample when downsampling total read counts to the indicated depths at 5 pg and 0.5 pg RNA inputs

In Fig. 4 the x axes of panels E and F were missing ‘2’ and the y axes contained a ‘$’ and “#” symbol after the values respectively.

In Fig. 6, in panel H the y axis contained a ‘$’ symbol after the reported values.

In Fig. 7, the x and y axis of panel C erroneously contained the ‘$’ symbol and in panel D both axes contained the “#” symbol.

The correct figures are presented in this Correction and the original article [1] has been corrected.

## References

[1] Jia E, Shi H, Wang Y (2021). Optimization of library preparation based on SMART for ultralow RNA-seq in mice brain tissues. BMC Genomics.

