# Supplementary material for: Optimization of library preparation based on SMART for ultralow RNA-seq in mice brain tissues
Source: BMC Genomics. 2021 Nov 10;22:809. doi: 10.1186/s12864-021-08132-w (PMC8579666; doi:10.1186/s12864-021-08132-w)
Supplement: Supplementary file 1 — Additional file 1: Table S1. Summary of ulRNA-seq. [file 12864_2021_8132_MOESM1_ESM.doc]

Table S1. Summary of ulRNA-seq.

| **Sample** | **Redas aligned to genome** | **Percent of aligned reads that map to RefSeq transcripts** | **Error Rate (%)** | **GC Content(%)** |
| --- | --- | --- | --- | --- |
| **TSO-rN-5pg-1** | 16421200 | 83.10% | 0.03 | 52.26 |
| **TSO-rN-5pg-2** | 15687983 | 83.61% | 0.03 | 51.91 |
| **TSO-rN-5pg-3** | 8224785 | 82.51% | 0.03 | 51.77 |
| **TSO-rU-5pg-1** | 15269862 | 87.48% | 0.04 | 45.91 |
| **TSO-rU-5pg-2** | 5796594 | 79.39% | 0.05 | 42.6 |
| **TSO-rU-5pg-3** | 11590327 | 77.42% | 0.04 | 41.72 |
| **TSO-rG-5pg-1** | 14160170 | 83.05% | 0.04 | 48.97 |
| **TSO-rG-5pg-2** | 12492695 | 84.84% | 0.03 | 48.59 |
| **TSO-rG-5pg-3** | 14225555 | 69.89% | 0.05 | 41.11 |
| **TSO-rN-0.5pg-1** | 3811623 | 24.94% | 0.08 | 42.59 |
| **TSO-rN-0.5pg-2** | 9893931 | 41.21% | 0.06 | 44.23 |
| **TSO-rN-0.5pg-3** | 10175669 | 29.24% | 0.05 | 46.95 |
| **TSO-rU-0.5pg-1** | 6646194 | 10.68% | 0.09 | 40.27 |
| **TSO-rU-0.5pg-2** | 5499332 | 0.85% | 0.12 | 44.15 |
| **TSO-rU-0.5pg-3** | 4087262 | 26.80% | 0.11 | 30.02 |
| **TSO-rG-0.5pg-1** | 15777583 | 22.50% | 0.16 | 21.98 |
| **TSO-rG-0.5pg-2** | 14247814 | 17.35% | 0.18 | 19.56 |
| **TSO-rG-0.5pg-3** | 8704063 | 28.22% | 0.15 | 20.29 |
