# Supplementary material for: Optimization of library preparation based on SMART for ultralow RNA-seq in mice brain tissues
Source: BMC Genomics. 2021 Nov 10;22:809. doi: 10.1186/s12864-021-08132-w (PMC8579666; doi:10.1186/s12864-021-08132-w)
Supplement: Supplementary file 2 — Additional file 2: Figs. S1. Compared the efficiency of different reverse transcriptase. Figs. S2. The number of genes and cell marker genes detected at 1 ng RNA inputs. Figs. S3. Gene body coverage averaged over different input amounts of RNA sequenced with the different reverse transcriptase library construction. Figs. S4. Principal component analysis and correlation analysis of normalized gene expression values for all samples RNA-Seq datasets analyzed. Figs. S5. Correlation analysis for all samples RNA-Seq datasets. Figs. S6. Comparison of differentially expressed alternative splicing (DEAS) under the different terminal modification TSO for 5 pg RNA inputs. Figs. S7. Gene body coverage averaged over the different structure of mRNA templates. Figs. S8. Comparison of differentially expressed alternative splicing (DEAS) under the different structures of mRNA templates for 5 pg RNA inputs. [file 12864_2021_8132_MOESM2_ESM.doc]

Fig. S1. Compared the efficiency of different reverse transcriptase. (A) (B) (C) (D) Yield of preamplified cDNA obtained using different reverse transcriptases for different input amounts of RNA. (E) For input amounts 5 pg RNA, the effects of five reverse transcriptases on Ct value of Hprt, 18S, and GAPDH. (F) For input amounts 0.5 pg RNA, the effects of five reverse transcriptase on Ct value of Hprt, 18S, and GAPDH.

Fig. S2. The number of genes and cell marker genes detected at 1 ng RNA inputs.

Fig. S3. Gene body coverage averaged over different input amounts of RNA sequenced with the different reverse transcriptase library construction. (A) Gene body coverage averaged over different reverse transcriptase and RNA input sequenced. (B) Heat maps of gene coverage of different reverse transcriptase and RNA input sequence.

Fig. S4. Principal component analysis and correlation analysis of normalized gene expression values for all samples RNA-Seq datasets analyzed. (A) Principal component analysis for all samples at 5 pg RNA inputs in different terminal modification TSO. (B) Principal component analysis for all samples at 0.5 pg RNA inputs in different terminal modification TSO. (C) Heatmap showing Pearson correlation of FPKM values (Blue indicates high correlation and red indicates low correlation).

Fig. S5. Correlation analysis for all samples RNA-Seq datasets. (A) Heatmap showing Pearson correlation between the three replicates for different TSO libraries at 5 pg RNA inputs. (B) Heatmap showing Pearson correlation between the three replicates for different TSO libraries at 0.5 pg RNA inputs.

Fig. S6. Comparison of differentially expressed alternative splicing (DEAS) under the different terminal modification TSO for 5 pg RNA inputs. ES: exon skipping, AD: alternative donor, AA: alternative adaptor, MXE: mutually exclusive exon, IR: intron retention.

Fig. S7. Gene body coverage averaged over the different structure of mRNA templates. (A) Heat maps of gene coverage of different structures of mRNA templates at 5 pg RNA input. (B) Heat maps of gene coverage of different structures of mRNA templates at 0.5 pg RNA input.

Fig. S8. Comparison of differentially expressed alternative splicing (DEAS) under the different structures of mRNA templates for 5 pg RNA inputs. ES: exon skipping, AD: alternative donor, AA: alternative adaptor, MXE: mutually exclusive exon, IR: intron retention.
